# Juvenile polyposis diagnosed with an integrated histological, immunohistochemical and molecular approach identifying new *SMAD4* pathogenic variants

**DOI:** 10.1007/s10689-022-00289-x

**Published:** 2022-01-25

**Authors:** Andrea Mafficini, Lodewijk A. A. Brosens, Maria L. Piredda, Cristian Conti, Paola Mattiolo, Giulia Turri, Maria G. Mastrosimini, Sara Cingarlini, Stefano F. Crinò, Matteo Fassan, Paola Piccoli, Michele Simbolo, Alessia Nottegar, Rita T. Lawlor, Alfredo Guglielmi, Aldo Scarpa, Corrado Pedrazzani, Claudio Luchini

**Affiliations:** 1 Department of Diagnostics and Public Health, Section of Pathology, University and Hospital Trust of Verona, 37134 Verona, Italy; 2 ARC-Net Research Center, University of Verona, Verona, Italy; 3 Department of Pathology, Utrecht, and Department of Pathology, University Medical Center Utrecht, Utrecht University, Radboud Institute for Molecular Life Sciences, Radboud University Medical Center, Nijmegen, The Netherlands; 4 Department of Surgical Sciences, Dentistry, Gynecology and Pediatrics, Unit of General and Hepatobiliary Surgery, University and Hospital Trust of Verona, 37134 Verona, Italy; 5 Department of Medicine, Section of Oncology, University and Hospital Trust of Verona, Verona, Italy; 6 Digestive Endoscopy Unit, The Pancreas Institute, University and Hospital Trust of Verona, Verona, Italy; 7 Surgical Pathology Unit, Department of Medicine (DIMED), University of Padua, and Veneto Institute of Oncology, IOV-IRCCS, Padua, Italy

**Keywords:** Familial juvenile polyposis syndrome, Juvenile polyp, Hamartomatous polyposis, *SMAD4*, Laparoscopic surgery, Germline variants

## Abstract

Juvenile polyposis (JP) is a rare familial syndrome characterized by the development of numerous hamartomatous polyps of the gastrointestinal tract and by an increased risk of developing gastrointestinal cancers. It follows a pattern of autosomal dominant inheritance and is associated with germline variants of *SMAD4* or *BMPR1A* genes. Differential diagnosis may be difficult based on histology alone, due to morphological similarities to other familial syndromes. Here we report a case of familial JP diagnosed in a 50-years woman with a familial history positive for gastrointestinal cancers and other tumor types. The patient presented with severe iron deficiency anemia and showed numerous polyps in the stomach and jejunum according to endoscopy and imaging. She underwent an intra-gastric laparoscopic removal of the major gastric polyp, followed by jejunal exploration and resection of a segment with multiple neoformations. Histological examination revealed the presence of hamartomatous polyposis. Gastric and intestinal samples were analyzed with next-generation sequencing. Molecular analysis showed that the patient harbored a germline splicing site variant of *SMAD4*, c.1139 + 3A > G, which was complemented by different somatic variants of the same gene in the different polyps. Immunohistochemistry for SMAD4 confirmed loss of protein expression in the polyps, with regular expression in normal cells. cDNA sequencing further confirmed the findings. We thus definitively diagnosed the woman as having JP thanks to an integrated approach based on histology, immunohistochemistry and molecular analysis. The identified variants, all previously reported as variants of unknown significance, were classified as pathogenic as they complemented each other leading to *SMAD4* loss.

**Supplementary Information:**

The online version contains supplementary material available at 10.1007/s10689-022-00289-x.

## Introduction

Hamartomas have been defined as an overgrowth of cells and tissues native to the anatomic site in which they arise [1, 2]. In the gastrointestinal system, hamartomas typically include both stromal and epithelial components [1–5]. Usually, they represent a solitary finding, but may also occur as part of a hamartomatous polyposis syndrome, such as juvenile polyposis syndrome (JP), Peutz-Jeghers syndrome (PJS), Cowden syndrome (CS), Cronkhite-Canada syndrome (CCS) or the Bannayan-Riley-Ruvalcaba syndrome (BRRS) [1–5]. Taken together, hamartomatous polyposis syndromes account for 1% of annual colorectal cancer cases, and their identification is crucial for patients and relatives for addressing appropriate screening and cancer-prevention strategies [5–9].

In the context of hamartomatous polyposis of the gastrointestinal tract, the two most frequent conditions are represented by JP and PJS [5]. Although the occurrence of sporadic juvenile polyps has been described, JP is a classic disorder following an autosomal-dominant inheritance. It is characterized by the presence of multiple hamartomatous polyps in the colorectum (> 90%), stomach (15%), duodenum (7%), and jejunum and ileum (7%) [4, 5, 10–12]. JP patients have a 50% increase in the lifetime risk of developing gastrointestinal cancers [5, 8, 10]. For instance, the incidence of JP-related colorectal cancer ranges from about 20% in patients under 35 years to almost 70% in the sixth decade [5, 13]. Loss of function of two key tumor suppressor genes belonging to the TGF-β signaling pathway, such as the bone morphogenetic protein receptor type 1A (*BMPR1A*) and the SMAD family member 4 (*SMAD4*), represents a well-established molecular pathogenetic mechanism of JP. *SMAD4* was the first gene reported to cause JP, and its germline variant is also associated with another condition, the hereditary hemorrhagic telangiectasia [14, 15]. Variants in *SMAD4* and *BMPR1A* account for about 18–20% of JP cases each, while the remaining 60% of cases are still orphan of a genetic driver [16–18]. Cases with *SMAD4* variants usually feature both gastric and intestinal polyps, and a large fraction displays high-grade adenomatous lesions and malformative vessels, while cases with *BMPR1A* variants feature only colorectal polyps with lack of adenomatous aspects and malformative vessels [16]. On the other hand, PJS is a rare hereditary condition characterized by muco-cutaneous pigmentation and hamartomatous polyps, predominantly affecting the small intestine [1–5, 19–21]. Like JP, PJS follows an autosomal dominant inheritance. It is caused by heterozygous germline pathogenic variants affecting the serine threonine kinase 11 tumor suppressor gene (*STK11* gene, also known as *LKB1*) [5, 22, 23]. This condition is associated with a wide clinical spectrum, which also includes bleeding, anemia, and obstructive symptoms. In adulthood, PJS is associated with an increased risk of developing different cancer types, including adenocarcinomas of the gastrointestinal tract [1–5, 7, 8]. This risk of developing cancer for patients with PJS increases with age, reaching a 80% risk at the age of 70 years [24]. Differential diagnosis between these entities and other rare conditions by histology alone is very challenging. Genetic tests for specific alterations may represent the decisive step to this end.

Of note, the correct identification of a genetic predisposition to hamartomatous polyposis, as well as a correct diagnosis among several distinctive potential entities, is becoming an urgent clinical issue with important implications for the affected patients and their relatives. Tailored surveillance should be offered to all patients with hamartomatous polyposis to manage their increased risk of cancer development. Furthermore, defining the inheritable gene alteration of each patient allows for screening and surveillance of their relatives. Here we report the paradigmatic case of a 50-years-old lady with hamartomatous polyposis with gastric and small-intestinal manifestations, diagnosed as JP based on the identification of novel pathogenic *SMAD4* variants. The histopathologic examination of the polyps, coupled with molecular profiling by next-generation sequencing, represents a very robust approach in this field.

## Materials and methods

### DNA extraction

Genomic DNA was extracted from formalin-fixed paraffin-embedded (FFPE) tissues using the GeneRead DNA FFPE kit (Qiagen) according to the manufacturer’s instructions. The kit procedure includes the removal of deaminated cytosine to prevent false results in DNA sequencing. Neoplastic cellularity was evaluated by two gastrointestinal pathologists (C.L., M.F.) on hematoxylin and eosin staining, and each tumor sample was manually microdissected with a fine-needle hypodermic syringe to enrich for tumor cells. Quantification of genomic DNA samples was performed with the Qubit dsDNA HS assay kit on a Qubit fluorometer (ThermoFisher) and qualification as previously described [25].

### Massive parallel sequencing (next-generation sequencing, NGS)

NGS was performed using the SureSelectXT HS CD Glasgow Cancer Core assay (www.agilent.com), hereinafter referred as CORE [26]. The panel spans 1.85 megabases of the genome and interrogates 174 genes for somatic variants, copy number alterations and structural rearrangements; the detail of targeted genes is reported in Supplementary Table 1. Sequencing libraries were prepared by targeted capture using the SureSelect kit (Agilent Technologies), with RNA baits targeting a bespoke set of selected genomic features. Briefly, 10–100 ng of genomic DNA extracted from FFPE tissue was enzymatically fragmented with the SureSelect Enzymatic Fragmentation Kit (Agilent Technologies). Overhanging DNA fragments were subsequently end-repaired, adenylated, ligated to indexing/sequencing adapters, enriched by PCR and purified following the manufacturer’s instructions. Sequencing was performed on a NextSeq 500 (Illumina) loaded with 2 captured library pools, using a high-output flow cell and 2 × 75 bp paired end sequencing.

CORE panel analysis started with demultiplexing performed with FASTQ Generation v1.0.0 on the BaseSpace Sequence Hub (https://basespace.illumina.com, last access 03/23/2021). Forward and reverse reads from each demultiplexed sample were aligned to the human reference genome (version hg38/GRCh38) using BWA and saved in the BAM file format [27]. BAM files were sorted, subjected to PCR duplicate removal, and indexed using biobam-bam2 v2.0.146 [28]. Coverage statistics were produced using samtools [29]. Calling of all variant types (small nucleotide variants, copy number variations and structural variants) was performed on tumor samples using a set of 20 non-neoplastic samples as a reference; these samples were retrieved at our institution, processed, and sequenced with the same workflow to yield comparable BAM files.

Single nucleotide variants were called using Shearwater [30]. Small (< 200 bp) insertions and deletions were called using Pindel [31]. Small nucleotide variants were further annotated using a custom pipeline based on vcflib (https://github.com/ekg/vcflib; last access 11/30/2020), SnpSift [32], the Variant Effect Predictor (VEP) software [33], and the NCBI RefSeq transcripts database (https://www.ncbi.nlm.nih.gov/refseq/; last access 11/30/2020). All candidate variants were manually reviewed using Integrative Genomics Viewer (IGV), version 2.9 [34], to exclude sequencing artefacts.

Tumor mutational burden and microsatellite instability were derived from sequencing analysis and computed following the method of Papke et al*.* [35]. Copy number alterations of targeted genes were detected using the geneCN software, developed at Wolfson Wohl Cancer Research Centre (https://github.com/wwcrc/geneCN; last access 10/31/2020). Structural rearrangements were detected using the BRASS software [36], and visually reviewed using Integrative Genomics Viewer (IGV), version 2.9 to exclude sequencing artefacts.

### Variant classification

Variants were classified following the five-tier classification system recommended by the joint consensus of the American College of Medical Genetics and Genomics and the Association for Molecular Pathology (ACMG/AMP) [37]. Variants were thus classified as Benign (class 1), Likely Benign (class 2), Variant of Uncertain Significance (VUS—class 3), Likely Pathogenic (class 4) and Pathogenic (class 5). Variants’ classification was retrieved from the ClinVar database when available (https://www.ncbi.nlm.nih.gov/clinvar/; last access 11/16/2020) and accepted when the record complied with the following requisites: reviewed by expert panel according to the ACMG/AMP guidelines and/or reported by multiple submitters with evaluation criteria according to the ACMG/AMP guidelines and no conflicts. When a consistent classification was unavailable or when the variant was not present in the ClinVar database, variants were evaluated in-house, according to the ACMG/AMP guidelines using also the following databases and software to gather and integrate all relevant information: My Cancer Genome (https://www.mycancergenome.org; last access 04/20/2020), Intogen [38] (https://www.intogen.org/search; last access 11/23/2020) and QIAGEN Clinical Insight (QCI) software (https://variants.qiagenbioinformatics.eu/qci/; last access 11/16/2020).

### Immunohistochemistry for SMAD4 (DPC4) protein

IHC was performed as a confirmation of the potential pathogenicity of the observed germline and somatic *SMAD4* variants. It was conducted on different inclusions deriving from both gastric and intestinal polyps and following a specific multi-step process, as already described [39, 40], Briefly, 4 μm, formalin-fixed and paraffin-embedded sections were immunostained with the following antibody, according to the manufacturer’s instructions: SMAD4 (clone: B-8; dilution: 1:1000; Santa Cruz, Dallas, TX, USA). Heat-induced antigen retrieval was performed using a heated plate and 0.01 mol/l of citrate buffer, pH 8.9, for 15 min. Light nuclear counterstaining was performed with hematoxylin. The samples were processed using a sensitive peroxidase-based “Bond polymer Refine” detection system in an automated Bond instrument (Vision-Biosystem, Leica, Milan, Italy). Sections incubated without the primary antibody served as negative controls. IHC were evaluated separately and in blind by two gastrointestinal pathologists (C.L., M.F.). The expression of the protein was evaluated at the nuclear level and as retained expression (immunostaining of the nuclei) or loss of expression (no immunostaining of the nuclei).

### Sanger sequencing of *SMAD4* mRNA in non-neoplastic tissue

Sanger sequencing was used to confirm the loss of heterozygosis of *SMAD4* mRNA in non-neoplastic cells of the patient due to the splice variant c.1139 + 3A > G. Three gastroduodenal samples from individuals not featuring the c.1139 + 3A > G variant and bearing a homozygous reference, a homozygous variant, and a heterozygous genotype for the rs140241965 and rs3819122 SNPs were used as assay controls. Fresh-frozen non-neoplastic gastroduodenal tissue samples were used for mRNA extraction using the AllPrep DNA/RNA kit (QIAGEN), according to the manufacturer’s protocol. Quality and quantity of the mRNA were assessed with the Qubit RNA BR Assay Kit on the Qubit fluorophore (ThermoFisher Scientific) and with the RNA ScreenTape Analysis kit on the Agilent tape station (Agilent Technologies) according to the manufacturer’s instructions. 1 μg of mRNA from each sample was reverse transcribed in a total reaction volume of 20 μl using the Superscript VILO kit and random hexamers (ThermoFisher Scientific) according to the manufacturer’s protocol. 2 μl of reverse transcription reaction were amplified using a primer pair that targets a 425 bp region in the 3′UTR of SMAD4 mRNA, corresponding to the genomic coordinates chr18:51,084,186–51,084,611 of the hg38 release of the human genome. The primers’ sequences are: FWD = CCACCCTCCTAAGTGGTGTG; REV = CCTTCTATCAATGACAAGCA GCC. The PCR mix was denatured for 5 min at 95 °C, and then 30 cycles of amplification were performed with the following thermal profile: 94 °C × 30 s, 60 °C × 45 s, 72 °C × 45 s. The PCR products were visualized with the D1000 ScreenTape kit on the Agilent tape station (Agilent Technologies) to confirm the presence of the proper molecular weight size products. PCR products were purified with Agen-court AMPure XP beads (Beckman Coulter) and labelled with BigDye® Terminator v3.1 (Applied Biosystems). Agencourt CleanSEQ magnetic beads (Beckman Coulter) were used for post-labeling DNA fragment purification, and sequence analysis was performed on the Applied Biosystems 3130xl Genetic Analyzer.

## Results

### Case presentation

**Fig. 1:**
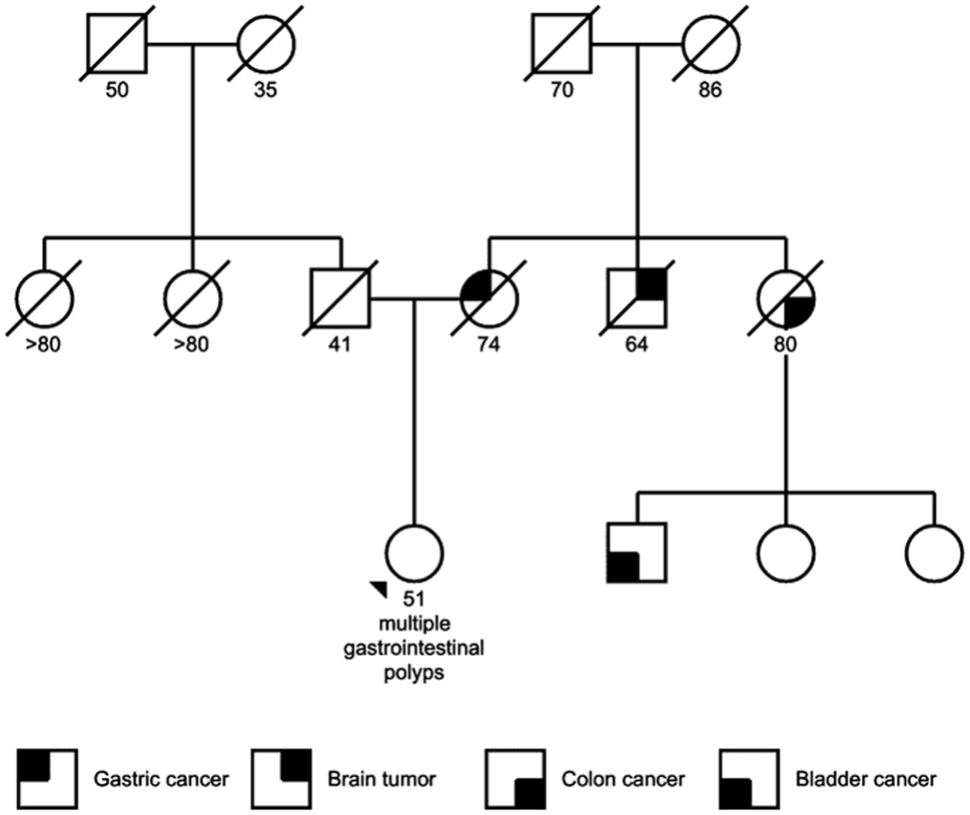
Family tree of the patient diagnosed with juvenile polyposis. Squares represent male and circles represent female subjects

**Fig. 2:**
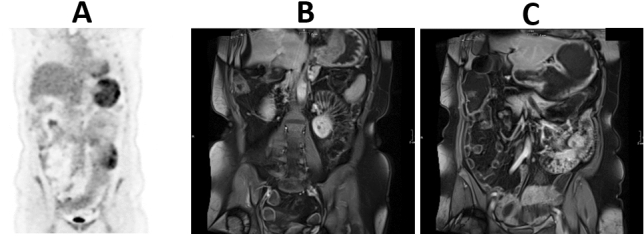
Pre-operative FDG-PET imaging shows an increased metabolic activity of the stomach with a radiotracer avid area (asterisk) in the larger curvature (SUV max 16.9). Hypermetabolic activity is also present in a small bowel loop in the left hypocondrium (arrowhead) (**A**). Magnetic resonance enterography shows increased enhancement of the gastric wall (**B**) and the first jejunal loop presents thickened walls and a hypervascularized mesentery with reactive lymph nodes (**C**)

A 50-years-old lady presenting to her local hospital for recurrent vomiting, anemia, protein-losing enteropathy, and weight loss (13 kg in 3 months), was referred to our Tertiary Care Center after an esophagogastroduodenoscopy (EGDS) evidenced multiple hamartomatous polyps and the presence of a large villous neoplasia, 11 cm in main diameter, located in the gastric fundus. The family tree was reconstructed (see Fig. 1, Progeny online pedigree tool, https://www.progenygenetics.com/online-pedigree, last accessed 06/18/2021). The patient’s mother died of gastric cancer, the maternal aunt died of colon cancer, the maternal uncle died of a brain tumor, while a maternal cousin suffered from bladder cancer. Laboratory tests revealed severe microcytic anemia (hemoglobin 6.4 g/dL, MCV 69.7 fl) and hypoalbuminemia (albumin 2.1 g/dL), whilst other parameters, among which B2 microglobulin, calcitonin, Anti-parietal cell antibodies, Castle's intrinsic factor, CEA, CA19-9, CA125, CA15-3, Cyfra 21–1, neuron specific enolase, were normal. Abdominal computed tomography (CT) confirmed the presence of an expansive, non-infiltrating lesion of the body and fundus of about 11 cm. The proximal small intestine appeared tense and strained, thickened for 13 cm. Fluorine 18-labeled fluorodeoxyglucose PET/CT scan (Fig. 2A) showed an uptake of the metabolic tracer at the upper third of the stomach, and at the level of first jejunal loops. Magnetic resonance enterography (MRE) (Fig. 2B) confirmed a tense gastric cavity with thickened body and fundus and jejunal thickening. Colonoscopy was normal except for left-sided diverticulosis. After anemia and hypoalbuminemia correction, the patient underwent surgery. Considering the uncertain diagnosis and the probably benign nature of the lesions, a laparoscopic approach was attempted. The gastric lesion was resected using a laparoscopic intra-gastric approach (Supplementary Fig. 1), as previously described in detail by our group [41, 42]. This multiport approach allowed to perform a full abdominal cavity exploration, an accurate intra-gastric port placement, the use of an endoscopic linear stapler for a complete full thickness tumor resection, and an easy and safe closure of gastrotomies. Furthermore, the jejunal exploration and resection with the subsequent anastomosis was successfully performed.

### Pathology report

**Fig. 3:**
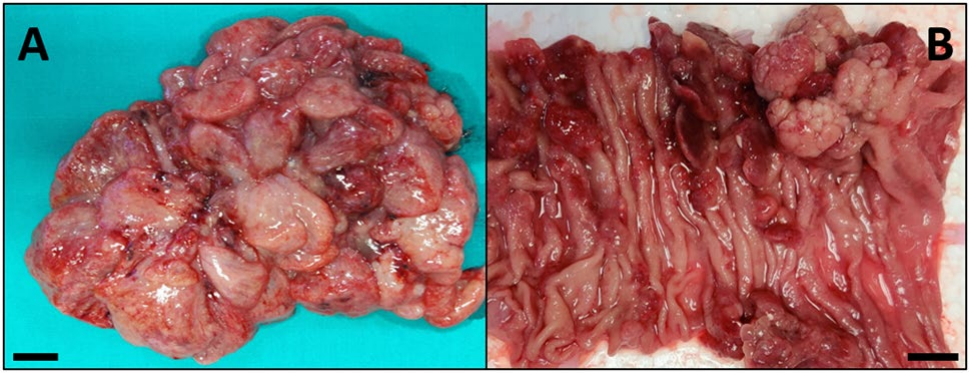
Macroscopic appearance of the resected specimens: gastric (**A**) and intestinal (**B**) polyps. Scale bar = 1 cm

Grossly, the gastric polyp was represented by a grey-brownish polypoid mass of 13 × 12 × 5 cm, with a jagged appearance, a soft-to-tight consistency, and an enlarged base of 5 × 4 cm (Fig. 3A). At the same time, the small bowel resection encompassed a tract of 13 cm of intestine, which harbored multiple polyps, with a diameter ranging from 3 mm to 4 cm, with a vaguely moriform surface (Fig. 3B).

**Fig. 4:**
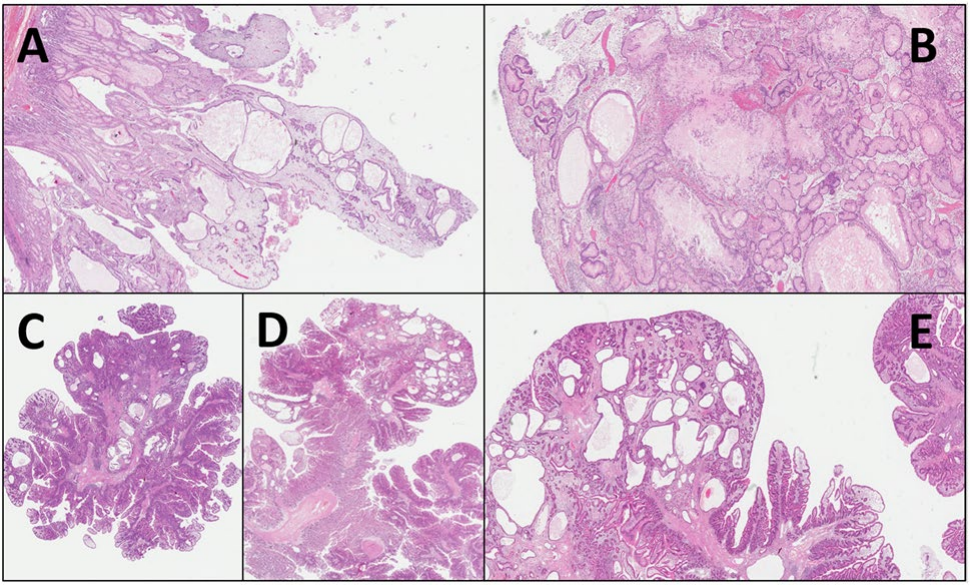
Histopathology of the polypoid lesions: gastric (A,B: Hematoxylin–eosin; **A** 4 × magnification, **B** 10 × magnification) and intestinal (**C**, **D**, **E** Hematoxylin–eosin; **C** 2 × magnification, **D** 4 × magnification, **E** 10 × magnification) polyps

Histological examination of the gastric polyp (Fig. 4A, B) showed predominant hamartomatous features, with a loose and edematous stroma comprising inflammatory cells. There were also marked aspects of foveolar hyperplasia, associated with several glandular ectasias, often of significant entity and such as to be classified as glandular cystic dilations. There were also hyperplastic-regenerative aspects of the foveolar epithelium, with focal reactive atypia. There were no foci of dysplasia, nor aspects of hyperplasia of the muscolaris mucosae. The histological examination of the resected tract of the small bowel (Fig. 4C, D, E) showed multiple polypoid lesions presenting histopathological features similar to each other and to what is observed at the gastric level. In particular, they showed a focally edematous stroma, comprising some inflammatory cells. There were marked aspects of intestinal villi hyperplasia, accompanied by focal glandular cystic ectasias. There were also some aspects of the “hyperplastic-serrated” type. Furthermore, there were focal areas showing low-grade dysplasia, and rare hyperplastic-regenerative aspects of the glandular epithelium were also noted. At last, there were only minimal aspects referable to hyperplasia of the muscolaris mucosae.

Globally considered, from the histopathologic point of view this case was diagnosed as a hamartomatous polyposis, suggesting the presence of a gastrointestinal polyposis syndrome. Based on morphological findings, the most reliable differential diagnoses were JP and PJS, but it is not possible to perform a definitive diagnosis based on histology alone. However, although at the gastric level it is very difficult to distinguish between JP and PJS, the small intestinal polyps resembled more the polyps in the course of JP, due to the presence of abundant edematous and inflammatory stroma, and nevertheless of cystically dilated crypts, while the typical features of polyps in the course of PJS, primarily an arborizing smooth muscle layer, were not predominantly present. On the other hand, the involvement of the small intestine is more typical in the case of PJS. In addition, most of the cases of JP also have polyps in the colon, but in this patient the colonoscopy was negative. Notably, it should be noted that JP associated with *SMAD4* variants might be predominant in the upper gastro-intestinal tract, as in this case. In the impossibility of further specifying the diagnosis based on the histopathological features, a multidisciplinary evaluation with clinical-pathological and genetic correlation became necessary.

### Molecular and immunohistochemical analysis

Molecular analysis was performed by next-generation sequencing (NGS) on histological samples of (a) the gastric polyp, (b) the largest intestinal polyp, and (c) normal intestine adjacent to polypoid lesions. The samples were sequenced using the CORE panel, which enables the detection of small nucleotide variants and structural alterations in 174 cancer-relevant genes, plus the evaluation of gross chromosomal alterations [26]. Results are summarized in Table 1. All three samples showed a heterozygous germline variant of *SMAD4* intron 9, involving the third base of the donor splicing site (c.1139 + 3A > G). The variant is recorded in dbSNP as a germline variant (rs786202607) and was never reported as a somatic variant. This variant was also reported twice in ClinVar (VCV000185983) as class 3 (variant of unknown significance) because it was absent from controls and predicted to alter exon splicing by multiple algorithms but lacked further evidence. However, the two previous ClinVar reports amount to three cases, which rise to four considering our patient. According to GnomAD data, this variant was never identified in a population of 76,111 subjects. We could not retrieve the exact number of subjects tested for JP, but a conservative estimate would be less than 20,000 considering the low prevalence of JP in the population. Under these conditions, the incidence of this variant was significantly higher in the population of patients compared to GnomAD controls (p = 0.0001), which would bring the variant to class 4 according to the ACMG/AMP criteria [37].

**Table 1:** Mutations identified in two different polyps (gastric and intestinal) and in a sample of normal small intestine from the same patient

Sample site	Gene	Alteration (cDNA)	Alteration (protein)	Allelic frequency (%)	ACMG Class^	Origin
	*SMAD4*	c.1139 + 3A > G	Splicing site	54	3, 4	Germline
Gastric polyp	*SMAD4*	c.1058A > G	p.Y353C	11	4	Somatic
	*SMAD4*	c.1610_1612delinsCCT	p.D537_E538delinsA*	7	4	Somatic
	*SMAD4*	c.1139 + 3A > G	Splicing site	63	3, 4	Germline
	*SMAD4*	c.425-6A > G	Splicing site	13	3, 4	Somatic
Intestinal polyp	*RNF43*	c.1111C > T	p.R371*	13	5	Somatic
	*RNF43*	c.182_183del	p.L61fs*13	20	4	Somatic
	*RNF43*	c.245del	p.L82*	12	4	Somatic
Normal intestine	*SMAD4*	c.1139 + 3A > G	Splicing site	54	3, 4	Germline

*SMAD4* Variants are annotated using RefSeq transcript NM_005359, RNF43 using RefSeq transcript NM_017763

^When two values are present, the first refers to the original classification reported in ClinVar, while the second to the new classification based on data of the present work

**Fig. 5:**
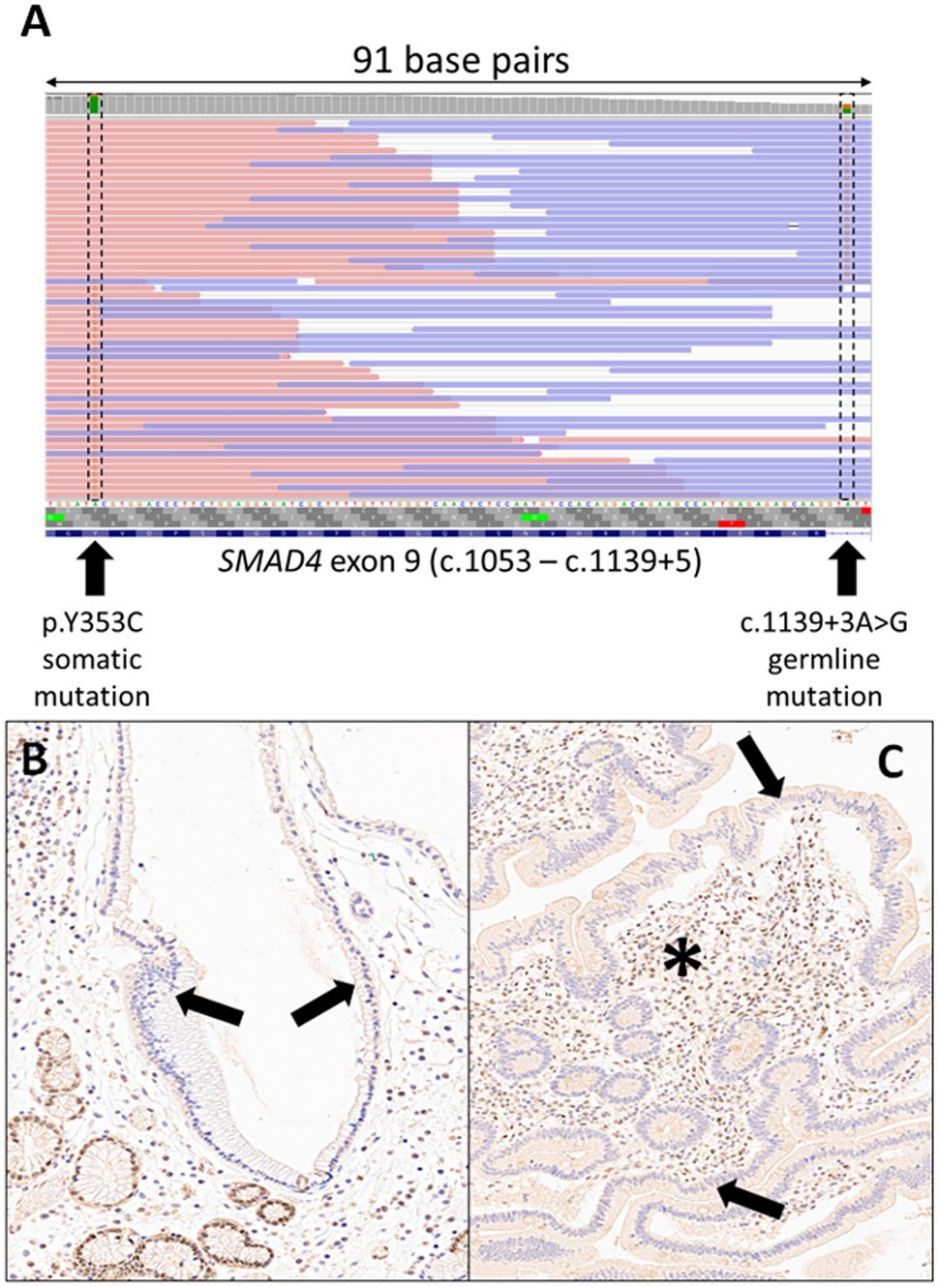
Somatic p.Y353C variant affecting the wild-type copy of *SMAD4* in a gastric polyp from a subject carrying the germline variant c.1139 + 3A > G. The alignment shows mutual exclusivity of the two alterations, which reside on either copy of chromosome 18 (**A**). Immunohistochemical analysis for SMAD4 in gastric (**B**) and intestinal (**C**) polyps shows SMAD4 loss of expression in epithelial cells (black arrows), with retained expression of the protein by stromal / inflammatory cells (asterisk, positive internal control)

The two polypoid lesions also harbored somatic variants that were not detected in the normal control sample. The gastric lesion displayed a somatic event near to the above reported germline splice variant (Table 1). This missense variant in the coding sequence of exon 9 (c.1058A > G; p.Y353C) was already reported both as a germline (dbSNP rs377767346; ClinVar VCV000597824) and somatic variant (Cosmic COSV61688013). As a germline variation, it was ranked as class 4 (likely pathogenic) for JP and hereditary hemorrhagic telangiectasia according to the ACMG guidelines [15]. As a somatic variant, it was demonstrated to promote epithelial to mesenchymal transition and the progression of disease in pancreatic adenocarcinoma [43]. Given the proximity with the germline splicing variant, it was possible to ascertain that the somatic mutational event hit the copy of the *SMAD4* gene which did not harbor the germline variant (Fig. 5A), thus confirming the alteration of both *SMAD4* copies in cells harboring both variants. A second somatic variant was detected at a lower allelic frequency (7%) in exon 12, a class 4 double base substitution resulting in a nonsense aminoacid change, p. D537_E538delinsA* that was never reported before. Given the distance from the other two variants, it was not possible to understand which copy of the *SMAD4* gene was affected by this somatic event. Moreover, the somatic variant could belong to a second clone of hyperplastic cells, as supported by the different allelic frequencies of the two somatic variants.

The largest intestinal polyp harbored one somatic *SMAD4* variant, affecting base -6 at the splice acceptor site of exon 4 (c.425-6A > G, Table 1). The variant is censored as germline (dbSNP rs377767327; Clinvar VCV000024803) and was never reported before as somatic. This variant was reported as a germline VUS (class 3) in a patient with JP (multiple gastric polyps) by the most recent Clinvar record (issued by GeneDx), while an older report by Aretz et al. identified this variant as pathogenic in a patient with a family history of JP [44]. The patient was diagnosed at 10 and underwent gastroscopy at 25, which led to the detection of numerous gastric polyps. Our report was the third of the same variation, the first as a somatic event. The variant was never detected in the control population of the GnomAD project and was predicted to alter mRNA splicing by multiple algorithms according to the Clinvar record and our annotation pipeline. In addition to the alteration of *SMAD4*, the intestinal sample featured three somatic pathogenic (class 4 or 5) *RNF43* variants, including two nonsense (p.L82*; p.R371*) and a frameshift (p.L61fs*13) variant. The two variants affecting codons 61 and 82 were near enough to verify if they were represented in the same DNA strand or not. Verification performed by visualization of the alignments with IGV confirmed that the two alterations were harbored by different DNA strands, meaning that these two nearby variants either arose in different clones or in different sister chromosomes of the same clone.

Considering that all the identified *SMAD4* variants were highly suspect for being pathogenic, but no conclusion could be done based on genetic data alone, we performed immunohistochemistry for SMAD4 to assess whether the compresence of the germline and somatic variants led to abolished protein expression. IHC clearly showed SMAD4 loss of expression in the epithelial cells of both the gastric and intestinal polyps, whereas inflammatory and stromal cells showed a retained expression of the protein in the nuclei (Fig. 5B, C). This analysis supported, at the protein level, the pathogenic nature of the reported *SMAD4* gene variants, including the germline c.1139 + 3A > G variant.

**Fig. 6:**
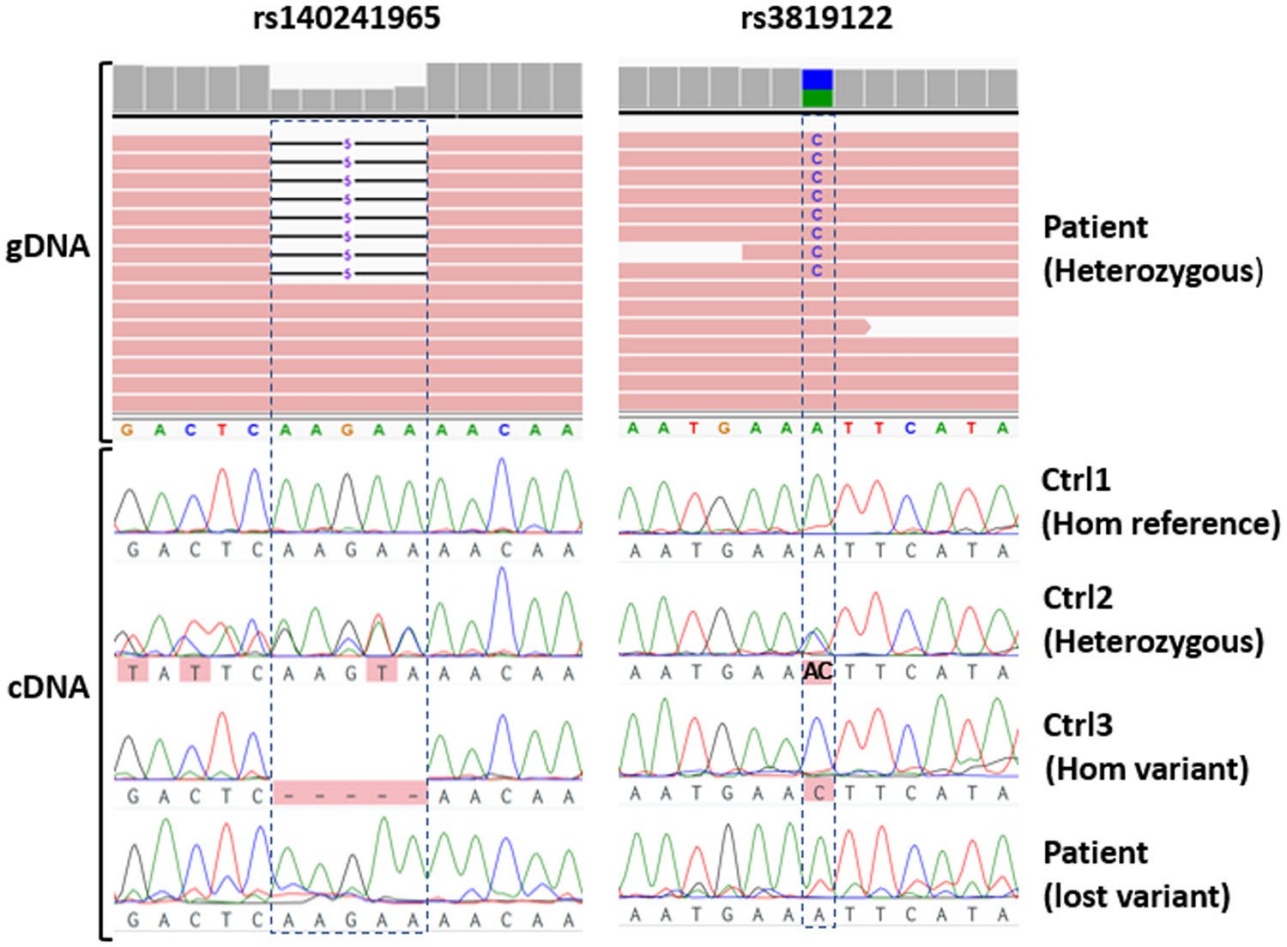
DNA Sequencing of a patient heterozygous for polymorphisms rs140241965, rs3819122 and the c.1139 + 3A > G splice site variant demonstrates loss of heterozygosis in the cDNA. All samples were from non-neoplastic gastroduodenal tissue. The upper panel shows the patient’s genomic DNA at the polymorphic sites. The lower panel shows cDNA sequencing. Ctrl1: cDNA of a control individual bearing none of the variants. Ctrl2: cDNA of a control individual bearing heterozygous rs140241965 and rs3819122 but not the splice site variant. Ctrl3: cDNA of a control individual bearing homozygous rs140241965 and rs3819122 but not the splice site variant. Patient: cDNA of the patient bearing heterozygous rs140241965 and rs3819122 and the splice site variant. Despite the patient has heterozygous genotype, the cDNA is homozygous, showing that the mature mRNA for one of the two alleles is not being produced

Given that the germline c.1139 + 3A > G variant was predicted to alter splicing, we sequenced the cDNA from normal gastroduodenal tissue of our patient to assess whether one of the two alleles was lost at the mRNA level. Our patient was heterozygous for two SNPs (rs140241965 and rs3819122) located in the 3’UTR of the *SMAD4* gene, downstream of the c.1139 + 3A > G variant. If the pre-mRNA containing the c.1139 + 3A > G variant was degraded due to abnormal splicing, the mRNA produced by our patient’s cells should result homozygous at those loci. As a further control we also sequenced the cDNA of three gastroduodenal samples from individuals not featuring the c.1139 + 3A > G variant and bearing a homozygous reference, a homozygous variant, and a heterozygous genotype for the above described SNPs (supplementary Fig. 2). The results (Fig. 6, supplementary Fig. 3) showed that, while the control subject with heterozygous genotype also had a heterozygous mRNA, our patient displayed a homozygous mRNA, supporting the loss of one of the alleles. Given that the c.1139 + 3A > G variant was the only difference in the *SMAD4* sequence of our patient compared to that of the heterozygous control subject, we concluded that the presence of this variant causes pre-mRNA degradation and thus we reclassified the variant from class 3 to class 4 (likely pathogenic) according to the ACMG/AMP criteria [37].

## Discussion

We herein presented a case of JP that was diagnosed and linked to pathogenic *SMAD4* variants by a combination of histology, next-generation sequencing (NGS), cDNA sequencing and immunohistochemistry. This integrative approach represents a robust model to improve routine diagnostic strategies as well as the management of patients with potential familial polyposis.

Juvenile polyps usually present in the first two decades of life, although at least 15% are found in adults as in our case [45]. Our patient was diagnosed at 50 years-old due to recurrent vomiting and protein-losing enteropathy, whereas diagnosis at earlier ages is prompted by lower intestinal bleeding and abdominal pain, caused by the formation of colorectal polyps [46]. The presence of gastric ad small intestinal polyps and iron dependent anemia are prominent features of *SMAD4*-dependent JP [16, 47], which amounts to about 20% of JP cases. Despite that, other syndromes may feature similar symptoms [1–5] and differential diagnosis would benefit by the integration of a genetic analysis. Next-generation sequencing was thus performed to exclude PJS or other syndromes resulting in the identification of a germline and three somatic variants in *SMAD4*, while no alterations were found in *STK11*, the PJS driver. While one somatic variant was a novel finding, the other three variants, including the germline one, were previously classified as variants of uncertain clinical significance due to lack of data. However, they were detected in patients featuring the upper gastrointestinal polyps characterizing *SMAD4*-dependent JP (ClinVar records VCV000185983, VCV000597824 and VCV000024803). Immunohistochemistry for SMAD4 showed that the somatic variants and the germline one complemented each other, leading to the complete loss of SMAD4 expression in the epithelial cells of the polyps, while inflammatory and stromal cells showed intact expression of the protein. Sequencing of cDNA from non-neoplastic gastroduodenal tissue of the patient showed that the presence of the germline variant caused loss of expression of one of the *SMAD4* alleles. Therefore, the integration of histology, NGS, cDNA sequencing and immunohistochemistry allowed to change the classification of the three variants from class 3 (unknown significance) to class 4 (probably pathogenic), especially that of the germline splice site variant c.1139 + 3A > G. This calls for surveillance and genetic testing of relatives of the JP patients bearing these variants, also considering the high risk of gastrointestinal malignancy displayed by *SMAD4*-JP patients [44, 48]. This was also consistent with the family tree of our patient: the mother died of gastric cancer and her maternal aunt died of colon cancer. While we could not verify the presence of the germline variant in these individuals, the maternal branch of the patient’s family tree seems to harbor a predisposition to cancer development which calls for surveillance [10, 48]. At the same time, we acknowledge that the absence of the second allele in normal tissue is no definitive proof of the pathogenicity of c.1139 + 3A > G, since a deep intronic variant that is not detectable in the sequencing assay may be missed. Together with the other evidence, however, it is likely that this variant (or at least the allele this variant resides on) is pathogenic.

While germline testing has been used in the past to profile probands for JP and other polyposis syndromes, the use of a multigene NGS panel and the simultaneous analysis of germline DNA and samples from the polypoid lesions was herein demonstrated to allow a better resolution of the pathogenetic mechanism, permitting the simultaneous identification of both germline and somatic alterations, whose effects could be verified by cDNA sequencing and immunolabeling of histological samples. This allowed not only to exclude other possible diseases, but also to address the patient’s treatment. Indeed, JP cases with *SMAD4* variants feature worse symptoms, including the frequent presence of high-grade adenomatous lesions and malformative vessels compared to cases with *BMPR1A* variants or no variants [16]. Since recurrence is often encountered in JP, perhaps a more radical or earlier approach may be suggested. As we speak, our patient was again treated to remove another large chunk of the stomach and duodenum due to the emergence of numerous polyps.

The application of a comprehensive NGS panel to polyposic lesions is also attractive when considering that roughly 60% of JP cases lack a driver germline alteration [16–18]. Indeed, the possibility to screen more than 100 genes at a time, detecting both small nucleotide and structural variations as in our case [26], would allow to directly ascertain the presence/absence of novel genetic alterations in each sample with only one assay. As an example of this scenario, we found additional *RNF43* variants in the intestinal polyp we analyzed. *RNF43* inactivating variants have been reported in several gastro-entero-pancreatic neoplasms, including colon adenocarcinoma, pancreatic adenocarcinoma, and intraductal papillary mucinous neoplasms of the pancreas [49–51]. *RNF43* is a ubiquitin E3 ligase that acts as a tumor suppressor via negative modulation of the Wnt pathway by targeting the frizzled receptor [52]. Its inactivation has been reported not only as a somatic event in tumors, but also as a germline alteration identified in a number of cases of serrated polyposis syndrome, thus defined as *RNF43*-associated serrated polyposis cases [53]. As for our patient, considering the concurrent presence of *SMAD4* variants, and the fact that *RNF43* was mutated solely in the intestinal sample, these somatic variants probably constituted a secondary event marking disease progression after the onset of the disease by *SMAD4* inactivation. However, since 60% of cases still have no driver, these finding still suggest how using a NGS panel could help find new clues in orphan cases at a reasonable cost compared to heavier genetic screening approaches.

In conclusion, we showed how a combined approach featuring the integration of histology, comprehensive targeted NGS and immunohistochemistry can allow the identification of germline and somatic alterations leading to the correct diagnosis of a hamartomatous polyposis; this case represents a next-generation model to approach patients with polyposis, even opening innovative horizons for the identification of new driver variants.

## Supplementary Information

Below is the link to the electronic supplementary material.

Supplementary Figure 1. Intra-gastric laparoscopic view of the polypoid lesion of the upper stomach (A). Intraoperative laparoscopic view of the first jejunal loop harboring multiple polypoid lesions (B) (TIF 510 kb)

Supplementary Figure 2. Genotype of three control individuals for the polymorphisms rs140241965 and rs3819122 located in the 3’UTR of the SMAD4 gene, as derived from deep sequencing of DNA from gastroduodenal non-neoplastic tissue. Ctrl1 is homozygous reference compared to the hg38 version of the human genome, Ctrl2 is heterozygous and Ctrl3 is homozygous variant (TIF 1781 kb)

Supplementary Figure 3. cDNA amplification of a 425bp region of SMAD4 3’UTR including polymorphisms rs140241965 (5bp deletion) and rs3819122 (A>C transversion) demonstrates loss of heterozygosis in the cDNA of a patient heterozygous for the polymorphisms and the c.1139+3A>G splice site variant. A) electropherogram of the amplicons, showing a longer amplicon for the wild-type allele and a shorter one for the variant allele. B) Densitometry of each electrophoretic band. Ctrl1: cDNA of a control individual bearing none of the variants. Ctrl2: cDNA of a control individual bearing heterozygous rs140241965 and rs3819122 but not the splice site variant. Ctrl3: cDNA of a control individual bearing homozygous rs140241965 and rs3819122 but not the splice site variant. Patient: cDNA of the patient bearing heterozygous rs140241965 and rs3819122 and the splice site variant. Despite the patient has heterozygous genotype, the cDNA only displays the longer amplicon for the wild-type allele, showing that the mature mRNA for the variant allele is not being produced (TIF 1164 kb)

Supplementary Table 1: Targeted genes in the CORE sequencing assay (PDF 42 kb)

## Data Availability

All data / information are available in the manuscript, related files and supplementary material.

## References

[1] Schreibman IR, Baker M, Amos C, McGarrity TJ (2005). The hamartomatous polyposis syndromes: a clinical and molecular review. Am J Gastroenterol.

[2] Manfredi M (2010). Hereditary hamartomatous polyposis syndromes: understanding the disease risks as children reach adulthood. Gastroenterol Hepatol (N Y).

[3] Kidambi TD, Kohli DR, Samadder NJ, Singh A (2019). Hereditary polyposis syndromes. Curr Treat Options Gastroenterol.

[4] Gilad O, Rosner G, Fliss-Isakov N (2019). Clinical and histologic overlap and distinction among various hamartomatous polyposis syndromes. Clin Transl Gastroenterol.

[5] International Agency for Research on Cancer (2019) WHO Classification of Tumours Editorial Board. Digestive system tumors.

[6] Spoto CPE, Gullo I, Carneiro F (2018). Hereditary gastrointestinal carcinomas and their precursors: an algorithm for genetic testing. Semin Diagn Pathol.

[7] Brosens LAA, Offerhaus GJA, Giardiello FM (2015). Hereditary colorectal cancer: genetics and screening. Surg Clin North Am.

[8] Vos S, van der Post RS, Brosens LAA (2020). Gastric epithelial polyps: when to ponder, when to panic. Surg Pathol Clin.

[9] Ma C, Giardiello FM, Montgomery EA (2014). Upper tract juvenile polyps in juvenile polyposis patients: dysplasia and malignancy are associated with foveolar, intestinal, and pyloric differentiation. Am J Surg Pathol.

[10] Brosens LAA, van Hattem A, Hylind LM (2007). Risk of colorectal cancer in juvenile polyposis. Gut.

[11] Latchford AR, Neale K, Phillips RKS, Clark SK (2012). Juvenile polyposis syndrome: a study of genotype, phenotype, and long-term outcome. Dis Colon Rectum.

[12] Wain KE, Ellingson MS, McDonald J (2014). Appreciating the broad clinical features of SMAD4 mutation carriers: a multicenter chart review. Genet Med.

[13] Shen N, Wang X, Lu Y (2020). Importance of early detection of juvenile polyposis syndrome. Medicine (Baltimore).

[14] Houlston R, Bevan S, Williams A (1998). Mutations in DPC4 (SMAD4) cause juvenile polyposis syndrome, but only account for a minority of cases. Hum Mol Genet.

[15] Shovlin CL, Simeoni I, Downes K (2020). Mutational and phenotypic characterization of hereditary hemorrhagic telangiectasia. Blood.

[16] Handra-Luca A, Condroyer C, de Moncuit C (2005). Vessels’ morphology in SMAD4 and BMPR1A-related juvenile polyposis. Am J Med Genet A.

[17] Howe JR, Sayed MG, Ahmed AF (2004). The prevalence of MADH4 and BMPR1A mutations in juvenile polyposis and absence of BMPR2, BMPR1B, and ACVR1 mutations. J Med Genet.

[18] Friedl W, Uhlhaas S, Schulmann K (2002). Juvenile polyposis: massive gastric polyposis is more common in MADH4 mutation carriers than in BMPR1A mutation carriers. Hum Genet.

[19] Peutz JLA (1921). Over een zeer merkwaardige, gecombineerde familiaire polyposis van de slijmvliezen van den tractus intestinalis met die van de neuskeelholte en gepaard met eigenaardige pigmentaties van huid-en slijmvliezen. Nederl Maandschr Geneesk.

[20] Jeghers H, McKusick VA, Katz KH (1949). Generalized intestinal polyposis and melanin spots of the oral mucosa, lips and digits. N Engl J Med.

[21] Tomlinson IP, Houlston RS (1997). Peutz-Jeghers syndrome. J Med Genet.

[22] Hemminki A, Markie D, Tomlinson I (1998). A serine/threonine kinase gene defective in Peutz-Jeghers syndrome. Nature.

[23] Jenne DE, Reimann H, Nezu J (1998). Peutz-Jeghers syndrome is caused by mutations in a novel serine threonine kinase. Nat Genet.

[24] Hearle N, Schumacher V, Menko FH (2006). Frequency and spectrum of cancers in the Peutz-Jeghers syndrome. Clin Cancer Res.

[25] Simbolo M, Gottardi M, Corbo V (2013). DNA qualification workflow for next generation sequencing of histopathological samples. PLoS ONE.

[26] Mafficini A, Lawlor RT, Ghimenton C (2021). Solid pseudopapillary neoplasm of the pancreas and abdominal desmoid tumor in a patient carrying two different BRCA2 germline mutations: new horizons from tumor molecular profiling. Genes.

[27] Li H, Durbin R (2009). Fast and accurate short read alignment with Burrows-Wheeler transform. Bioinformatics.

[28] Tischler G, Leonard S (2014). biobambam: tools for read pair collation based algorithms on BAM files. Source Code Biol Med.

[29] Li H, Handsaker B, Wysoker A (2009). The sequence alignment/map format and SAMtools. Bioinformatics.

[30] Gerstung M, Papaemmanuil E, Campbell PJ (2014). Subclonal variant calling with multiple samples and prior knowledge. Bioinformatics.

[31] Ye K, Schulz MH, Long Q (2009). Pindel: a pattern growth approach to detect break points of large deletions and medium sized insertions from paired-end short reads. Bioinformatics.

[32] Cingolani P, Patel VM, Coon M (2012). Using drosophila melanogaster as a model for genotoxic chemical mutational studies with a new program. SnpSift Front Genet.

[33] McLaren W, Pritchard B, Rios D (2010). Deriving the consequences of genomic variants with the Ensembl API and SNP Effect Predictor. Bioinformatics.

[34] Robinson JT, Thorvaldsdóttir H, Winckler W (2011). Integrative genomics viewer. Nat Biotechnol.

[35] Papke DJ, Nowak JA, Yurgelun MB (2018). Validation of a targeted next-generation sequencing approach to detect mismatch repair deficiency in colorectal adenocarcinoma. Mod Pathol.

[36] Ahdesmäki MJ, Chapman BA, Cingolani P (2017). Prioritisation of structural variant calls in cancer genomes. PeerJ.

[37] Richards S, Aziz N, Bale S (2015). Standards and guidelines for the interpretation of sequence variants: a joint consensus recommendation of the American College of Medical Genetics and Genomics and the Association for Molecular Pathology. Genet Med.

[38] Gundem G, Perez-Llamas C, Jene-Sanz A (2010). IntOGen: integration and data mining of multidimensional oncogenomic data. Nat Methods.

[39] Luchini C, Pea A, Lionheart G (2017). Pancreatic undifferentiated carcinoma with osteoclast-like giant cells is genetically similar to, but clinically distinct from, conventional ductal adenocarcinoma. J Pathol.

[40] Luchini C, Parcesepe P, Nottegar A (2016). CD71 in gestational pathology: a versatile immunohistochemical marker with new possible applications. Appl Immunohistochem Mol Morphol.

[41] Pedrazzani C, Vitali M, Guglielmi A (2015). A case of unexpected gastric mass. JAMA Surg.

[42] Rivelli M, Fernandes E, Conti C (2021). Laparoscopic intragastric resection of gastric synovial sarcoma: report of the first ever case with video demonstration. World J Surg Oncol.

[43] Wang Z, Li Y, Zhan S (2019). SMAD4 Y353C promotes the progression of PDAC. BMC Cancer.

[44] Aretz S, Stienen D, Uhlhaas S (2007). High proportion of large genomic deletions and a genotype phenotype update in 80 unrelated families with juvenile polyposis syndrome. J Med Genet.

[45] Gorlin RJ, Cohen MM, Condon LM, Burke BA (1992). Bannayan-Riley-Ruvalcaba syndrome. Am J Med Genet.

[46] Adolph VR, Bernabe K (2008). Polyps in children. Clin Colon Rectal Surg.

[47] Honda Y, Sato Y, Yokoyama J (2013). Familial juvenile polyposis syndrome with a novel SMAD4 germline mutation. Clin J Gastroenterol.

[48] Howe JR, Mitros FA, Summers RW (1998). The risk of gastrointestinal carcinoma in familial juvenile polyposis. Ann Surg Oncol.

[49] Yaeger R, Chatila WK, Lipsyc MD (2018). Clinical sequencing defines the genomic landscape of metastatic colorectal cancer. Cancer Cell.

[50] Cancer Genome Atlas Research Network (2017). Integrated genomic characterization of pancreatic ductal adenocarcinoma. Cancer Cell.

[51] Amato E, dal Molin M, Mafficini A (2014). Targeted next-generation sequencing of cancer genes dissects the molecular profiles of intraductal papillary neoplasms of the pancreas. J Pathol.

[52] Koo B-K, Spit M, Jordens I (2012). Tumour suppressor RNF43 is a stem-cell E3 ligase that induces endocytosis of Wnt receptors. Nature.

[53] Valle L, de Voer RM, Goldberg Y (2019). Update on genetic predisposition to colorectal cancer and polyposis. Mol Aspects Med.

