# Supplementary figures and images for: Juvenile polyposis diagnosed with an integrated histological, immunohistochemical and molecular approach identifying new SMAD4 pathogenic variants
Source: Fam Cancer. 2022 Jan 25;21(4):441–51. doi: 10.1007/s10689-022-00289-x (PMC9636285; doi:10.1007/s10689-022-00289-x)

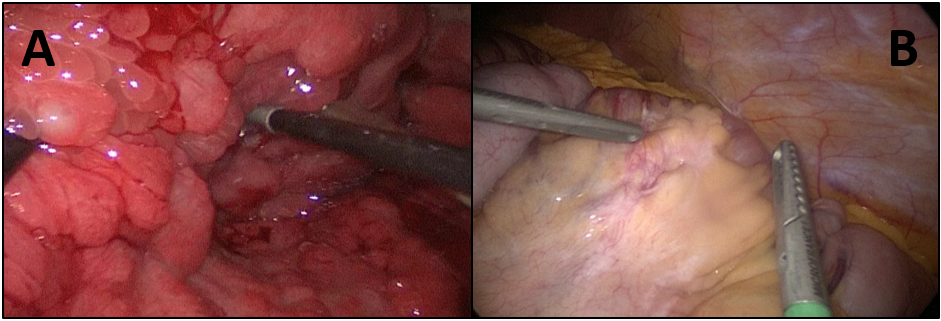

Supplement: Supplementary file 1 — Supplementary Figure 1. Intra-gastric laparoscopic view of the polypoid lesion of the upper stomach (A). Intraoperative laparoscopic view of the first jejunal loop harboring multiple polypoid lesions (B) (TIF 510 kb) [file 10689_2022_289_MOESM1_ESM.tif]

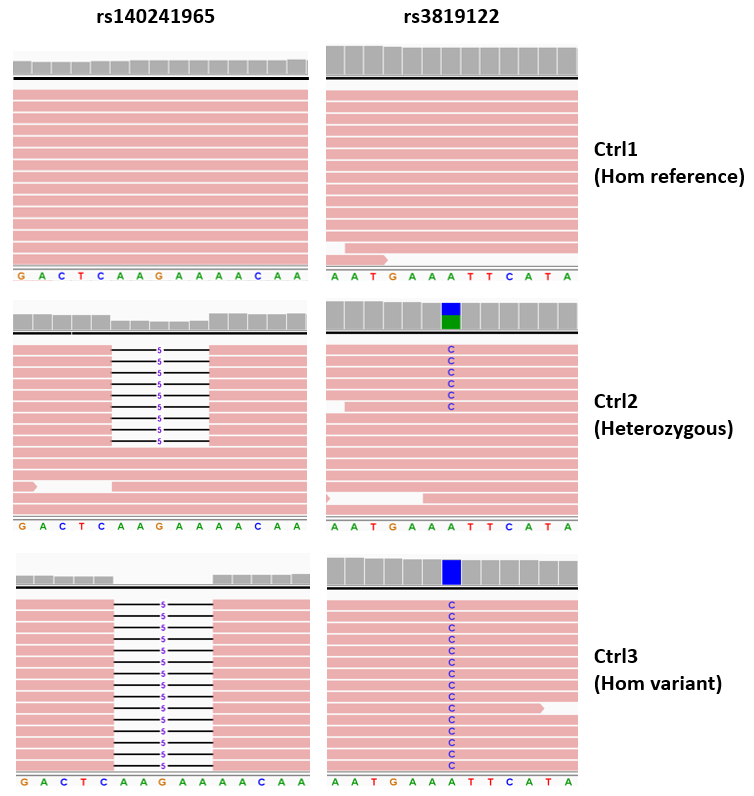

Supplement: Supplementary file 2 — Supplementary Figure 2. Genotype of three control individuals for the polymorphisms rs140241965 and rs3819122 located in the 3’UTR of the SMAD4 gene, as derived from deep sequencing of DNA from gastroduodenal non-neoplastic tissue. Ctrl1 is homozygous reference compared to the hg38 version of the human genome, Ctrl2 is heterozygous and Ctrl3 is homozygous variant (TIF 1781 kb) [file 10689_2022_289_MOESM2_ESM.tif]

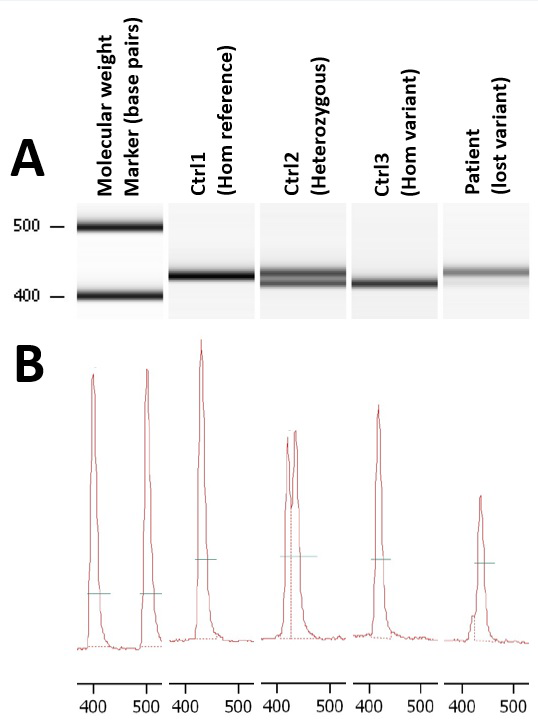

Supplement: Supplementary file 3 — Supplementary Figure 3. cDNA amplification of a 425bp region of SMAD4 3’UTR including polymorphisms rs140241965 (5bp deletion) and rs3819122 (A>C transversion) demonstrates loss of heterozygosis in the cDNA of a patient heterozygous for the polymorphisms and the c.1139+3A>G splice site variant. A) electropherogram of the amplicons, showing a longer amplicon for the wild-type allele and a shorter one for the variant allele. B) Densitometry of each electrophoretic band. Ctrl1: cDNA of a control individual bearing none of the variants. Ctrl2: cDNA of a control individual bearing heterozygous rs140241965 and rs3819122 but not the splice site variant. Ctrl3: cDNA of a control individual bearing homozygous rs140241965 and rs3819122 but not the splice site variant. Patient: cDNA of the patient bearing heterozygous rs140241965 and rs3819122 and the splice site variant. Despite the patient has heterozygous genotype, the cDNA only displays the longer amplicon for the wild-type allele, showing that the mature mRNA for the variant allele is not being produced (TIF 1164 kb) [file 10689_2022_289_MOESM3_ESM.tif]
